# Cytokine Reduction in the Setting of an ARDS-Associated Inflammatory Response with Multiple Organ Failure

**DOI:** 10.1155/2016/9852073

**Published:** 2016-01-17

**Authors:** Karl Träger, Christian Schütz, Günther Fischer, Janpeter Schröder, Christian Skrabal, Andreas Liebold, Helmut Reinelt

**Affiliations:** ^1^Department of Cardiac Anesthesia, University Hospital Ulm, 89070 Ulm, Germany; ^2^Clinic for Cardiothoracic and Vascular Surgery, University Hospital Ulm, 89070 Ulm, Germany

## Abstract

A 45-year-old male was admitted to our hospital with a small bowel obstruction due to torsion and was immediately scheduled for surgical intervention. At anesthesia induction, the patient aspirated and subsequently developed a severe SIRS with ARDS and multiple organ failure requiring the use of ECMO, CRRT, antibiotics, and low dose steroids. Due to a rapid deterioration in clinical status and a concurrent surge in inflammatory biomarkers, an extracorporeal cytokine adsorber (CytoSorb) was added to the CRRT blood circuit. The combined treatment resulted in a rapid and significant reduction in the levels of circulating inflammatory mediators. This decrease was paralleled by marked clinical stabilization of the patient including a significant improvement in hemodynamic stability and a reduced need for norepinephrine and improved respiratory function as measured by PaO_2_/FIO_2_, ventilator parameters, lung mechanics, and indirect measures of capillary leak syndrome. The patient could be discharged to a respiratory weaning unit where successful respiratory weaning could be achieved later on. We attribute the clinical improvement to the rapid control of the hyperinflammatory response and the reduction of inflammatory mediators using a combination of CytoSorb and these other therapies. CytoSorb treatment was safe and well tolerated, with no device-related adverse effects observed.

## 1. Introduction

Pulmonary aspiration of gastric or oropharyngeal fluid is a serious complication of general anesthesia that can lead to the development of acute respiratory distress syndrome (ARDS) in up to 30% of patients, the need for extracorporeal membrane oxygenation (ECMO), and the high risk of mortality [1]. Patients often develop a severe systemic inflammatory response syndrome (SIRS) that can lead to hemodynamic instability and multiple organ dysfunction syndrome (MODS), further complicating treatment [2]. A recently introduced extracorporeal cytokine hemoadsorption device called CytoSorb (CytoSorbents Corporation, USA) has gained interest in the field of critical care and cardiac surgery as a strategy to help control severe SIRS. Unlike metabolic approaches to anti-inflammation, CytoSorb is designed to directly capture and reduce mid-molecular weight inflammatory mediators (~10–60 kDa) in blood including both pro- and anti-inflammatory cytokines, chemokines, and bacterial exotoxins [3–5]. It works with standard hemodialysis or CRRT machines to purify blood extracorporeally. The technology is based on highly porous, biocompatible nonpolar polymer beads that are capable of capturing these inflammatory mediators from whole blood through size exclusion and nonspecific surface adsorption throughout the entire bead. The device is concentration dependent, reducing substances at high concentration efficiently, while having significantly less activity on substances at low concentration. This presumably is partially responsible for the observed reduction, but not complete elimination (which would be undesirable), of cytokines and other inflammatory mediators in patients treated with CytoSorb.

We herein report on a patient with an extreme SIRS response, cytokine storm, ARDS, and severe hemodynamic impairment following pulmonary aspiration during anesthesia induction, who was successfully treated with a combination of hemoadsorption with CytoSorb, ECMO, CRRT, antibiotics, and low dose steroids.

## 2. Case Presentation

**Figure 1:**
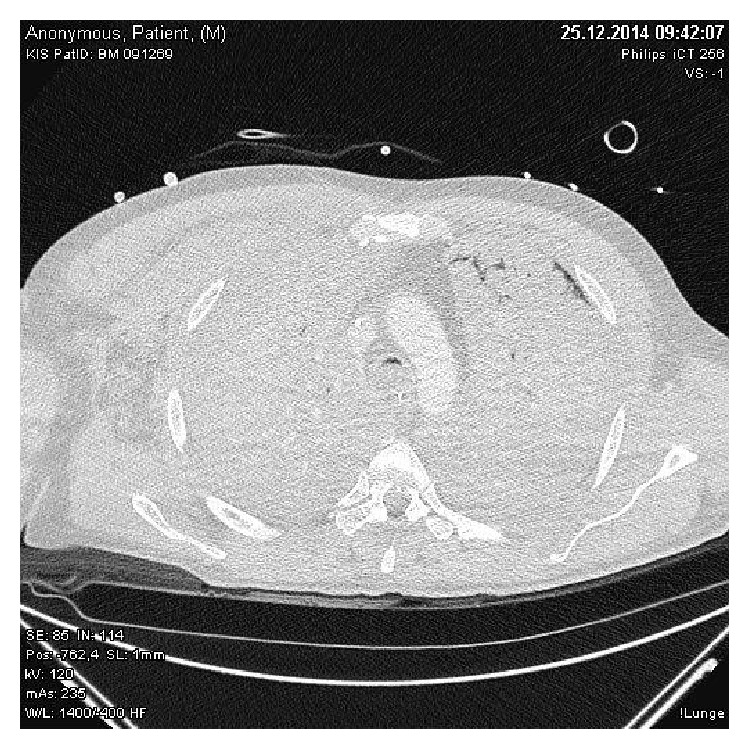
Lung computer tomography confirmed severe bilateral pulmonary consolidation with only minor residual ventilated lung areas.

A 45-year-old male patient was admitted to our hospital with a small bowel obstruction due to torsion and was immediately scheduled for surgical intervention. Prior medical history included an anterior rectal resection requiring an enterostomy due to anastomotic insufficiency. At anesthesia induction, the patient aspirated and immediately underwent bronchoscopy which confirmed pulmonary aspiration. Laparotomy was performed and decompression of the small bowel was achieved. However, during the ongoing operation (4 hours in total), the patient developed progressive and severe respiratory failure (paO_2_ 48 mmHg, FIO_2_ 1,0, paO_2_/FIO_2_ ratio 48, and paCO_2_ 75 mmHg, pH 7.09) despite mechanical ventilatory support. Therefore, the patient was switched to venovenous ECMO (VV-ECMO). However, four hours later, insufficient VV-ECMO flow and the rapid development of right ventricular failure necessitated a switch to central venoarterial ECMO (VA-ECMO). Sufficient ECMO flow was restored with an improvement in gas exchange (paO_2_ 83 mmHg, paCO_2_ 38 mmHg, pH 7.39). Twelve hours after the start of VA-ECMO, lung computer tomography confirmed severe bilateral pulmonary consolidation (see Figure 1).

However, the patient's condition continued to worsen with clinical and functional signs of severe exudative ARDS with alveolar edema (paO_2_ 49 mmHg, paCO_2_ 43 mmHg, and lung compliance <15 mL/cm H_2_O), systemic vasoplegia (norepinephrine dose 0.31 *µ*g/kg/min), and marked capillary leak syndrome (fluid balance + 4,080 mL after 12 hours in the ICU). In addition, the patient exhibited leukocytopenia (0.2 Giga/L), requiring the application of the granulocyte colony-stimulating factor analog filgrastim (Neupogen, Amgen) and acute kidney injury (AKIN Stage 3) necessitating continuous renal replacement therapy (CRRT-CVVHD, multiFiltrate, Fresenius Medical Care) [6]. At this time, the patient exhibited a massive increase in inflammatory biomarkers (IL-6 20,000 pg/mL, IL-8 24,656 pg/mL, and PCT > 100 *µ*g/L). Microbiological findings of intraoperative abdominal swabs revealed* Klebsiella*,* E. coli*, and Enterococci, while material from the respiratory tract indicated growth of* E. coli* and* Candida*. Combined treatment with meropenem (primed continuous infusion), linezolid, and anidulafungin was initiated. Further treatment included bronchoscopy, continued VA-ECMO combined with protective ventilation, kinetic positioning using a Rotorest (KCI) bed, and application of the sepsis bundle including administration of low dose hydrocortisone.

**Figure 2:**
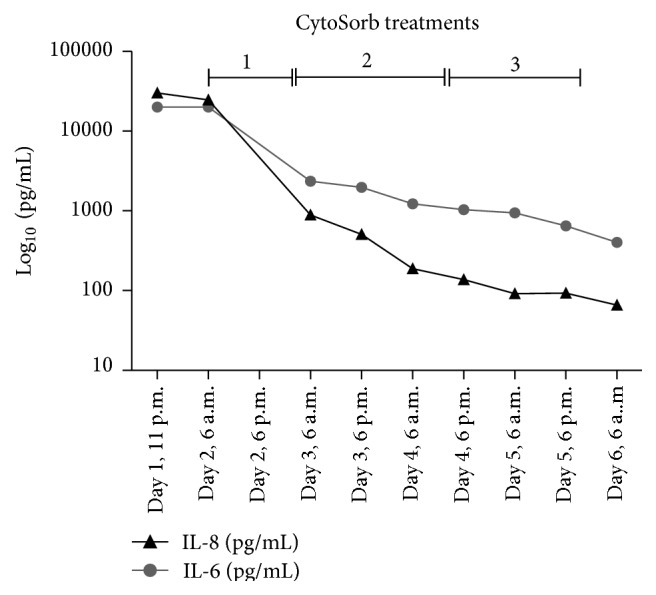
Course of IL-6 and IL-8 throughout the three treatments with the CytoSorb hemoadsorption device. Note that the *y*-axis is depicted in log⁡10 scale.

**Figure 3:**
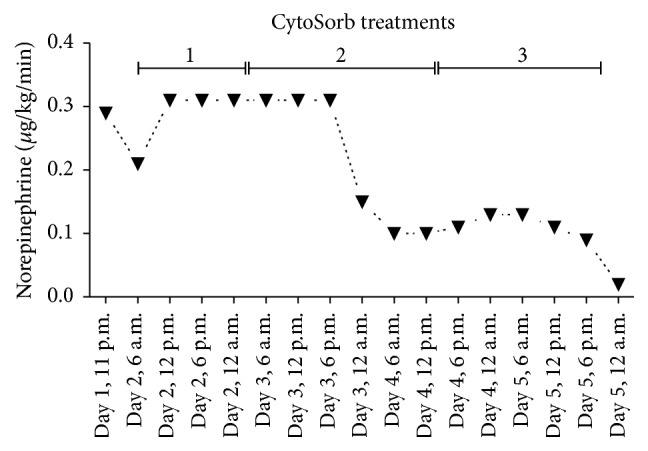
Need for norepinephrine throughout the treatment period.

**Figure 4:**
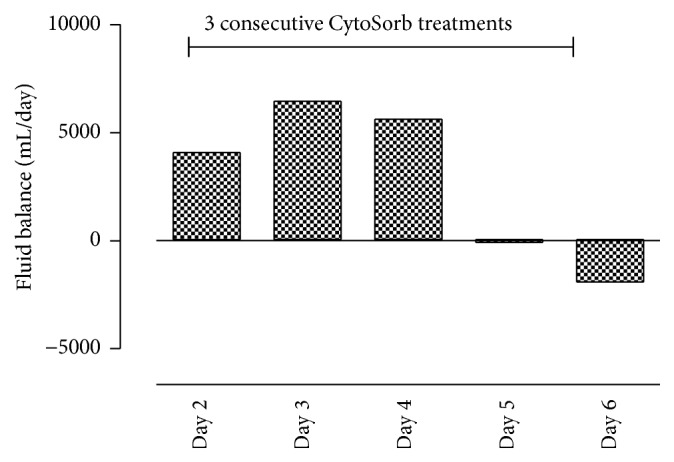
Fluid balance during the three consecutive CytoSorb treatments.

Despite these interventions, the patient remained in septic shock and was in critical status with regard to vasoplegia, hemodynamics, and capillary leakage. On post-op Day 1, an extracorporeal cytokine hemoadsorption cartridge (CytoSorb, CytoSorbents Corp., USA) was added in series to the CRRT system (Fresenius multiFiltrate) in a predialyzer position. During CytoSorb treatment, antibiotics were administered routinely as follows: meropenem 500 mg bolus followed by infusion of 1.45 mg/kg/h, anidulafungin prolonged infusion at 200 mg per day, and linezolid prolonged infusion 600 mg twice a day. A total of three treatments with a combination of CytoSorb and CRRT were consecutively performed for a total of 85 hours (20 hours + 35 hours + 29 hours) at a blood flow rate of 100–140 mL/min. Regional anticoagulation was achieved using a citrate-based protocol. Of note, the first treatment was interrupted for 4.5 hours due to a thoracic CT scan and surgical revision in order to reposition the arterial ECMO cannula to improve blood flow. When treatment was paused, the blood circuit was flushed with saline and was then later safely restarted. Following the addition of CytoSorb, a pronounced decrease in the concentrations of IL-6 and IL-8 was observed which continued to decrease further in the following days (see Figure 2). As shown in Figure 2, when the patient was on CRRT and VA-ECMO alone on Day 0, prior to CytoSorb usage, there was no change in the plasma levels of either IL-6 and IL-8. This cytokine reduction was associated temporally with a stabilization of clinical parameters. The patient stabilized hemodynamically under continued CytoSorb, VA-ECMO, and CRRT treatment, and the need for norepinephrine was significantly reduced (Figure 3). In addition, respiratory function improved during the treatment course, with a disappearance of any signs of alveolar exudation as confirmed by daily bronchoscopy. In parallel, the severity of capillary leakage as demonstrated by daily fluid needs and daily fluid balance became less apparent (Figure 4).

At postoperative Day 12, therapy was started with methylprednisolone following the Meduri protocol, in order to inhibit fibroproliferation in the lung and risk of fibrosis during ARDS [7]. Gradual additional improvements in lung recruitment and alveolar ventilation were observed. A percutaneous tracheostomy was performed on Day 13. By postoperative Day 18, the patient's respiratory function, along with gas exchange and lung mechanics, on mechanical ventilation had sufficiently improved such that VA-ECMO was discontinued. CRRT had to be continued for a period of 20 days and could then be stopped after sufficient recovery of renal function.

On Day 27, the patient was transferred to a respiratory weaning unit where the patient was subsequently successfully weaned off mechanical ventilation, with a discontinuation of CRRT and the recovery of renal function.

Informed consent to use clinical data for scientific purposes was obtained from the patient's legal caregiver.

## 3. Discussion

This case report highlights the stabilization and successful treatment of a complicated pulmonary aspiration postsurgical patient with septic shock and polymicrobial infection, severe exudative ARDS, renal failure, and a severe SIRS response with pronounced hypercytokinemia. Treatment encompassed a combination of cytokine reduction and inflammation reduction with CytoSorb, extracorporeal oxygenation and support with VA-ECMO, renal support with CRRT, low dose hydrocortisone treatment per sepsis bundle guidelines, and infection source control with broad spectrum antibiotics. Underlying the multiple organ failure was a severe SIRS/sepsis response, despite initial leukopenia, with extremely high levels of IL-6 (20,000 pg/mL) and IL-8 (24,656 pg/mL), exudative alveolar edema, systemic vasoplegia, and marked capillary leak syndrome. The use of a CytoSorb cytokine adsorber with this multipronged therapeutic approach resulted in a profound decrease in plasma concentrations of both IL-6 and IL-8, both classic inflammatory mediators. This reduction was paralleled by a significant clinical stabilization of the patient during the course of the combined treatment. Importantly, the treatment bundle was able to reverse the systemic vasoplegia and hemodynamic collapse, which is unlikely to be fully explained by the limited circulatory support of central VA-ECMO. In addition, the combined treatment reversed the highly exudative phase of ARDS, as demonstrated by bronchoscopy, and appeared to decrease capillary leak syndrome and alveolar fluid accumulation, as evidenced by improved respiratory function, a significantly reduced need for fluid resuscitation, and achievement of a negative fluid balance shortly after therapy initiation. Although a causal relationship cannot be proven, we presume that controlling the excessive inflammatory response helped to stabilize the patient. These observations are in line with a number of recently published case reports using CytoSorb in septic patients, correlating the positive effects of cytokine adsorption on inflammation, hemodynamics, and organ recovery [3, 4].

More randomized controlled studies using CytoSorb in critically ill patients will help to establish the true benefit of the therapy. Certainly, this case report that uses multiple interventions to treat this complex patient prevents us from ascribing the patient's positive outcome to any one treatment modality. In addition, cytokine levels could have gradually decreased on their own due to the use of antibiotics (e.g., improved source control reduces stimulation of the immune response), as was observed in the largest community acquired pneumonia and sepsis study (GenIMS) [2], or low dose hydrocortisone (administered here according to sepsis bundle guidelines) which has been previously associated with reduced concentrations of IL-6 and diminished* de novo* production of several interleukins [8]. We would note, however, that none of these approaches are mutually exclusive. In fact, they may all be synergistic with each other and may be needed to achieve the elusive improvements in mortality that no single sepsis treatment has yet been able to demonstrate or maintain.

In conclusion, we believe that control over the patient's initial hyperinflammatory response was a key element in helping clinically stabilize the patient, allowing for organ recovery and ultimately survival. In this very challenging clinical case, we used all of the tools available to us to do so, including CytoSorb cytokine adsorption, CVVH, VA-ECMO, antibiotics, and steroids. CytoSorb is the newest therapy in our toolkit and was safe and well tolerated with no device-related adverse events and easy to implement as part of the CVVH circuit. Based upon our positive clinical experience with CytoSorb as an adjunctive therapy to standard of care therapy for critical illnesses to date, we look forward to future systematic clinical trials that will clarify its contribution and potential in the treatment of severe sepsis and septic shock.
